# Initial presentation with elevated transaminases and subsequent hematuria in limb-girdle muscular dystrophy type 2B: A case report

**DOI:** 10.1097/MD.0000000000046926

**Published:** 2026-01-09

**Authors:** Zhenhua Ji, Hua Zheng, Ying Yang, Dan Li, Zhong Li, Xinxin Guo

**Affiliations:** a Department of Nephrology, Central Hospital Affiliated to Shenyang Medical College, Shenyang, China; b Department of Pharmacy, Central Hospital Affiliated to Shenyang Medical College, Shenyang, China; c Department of Pharmacy, Dalian Women and Children’s Medical Center (Group), Dalian, China.

**Keywords:** hematuria, limb-girdle muscular dystrophy type 2B, transaminase

## Abstract

**Rationale:**

Limb-girdle muscular dystrophy type 2B (LGMD2B) is a degenerative muscle disorder induced by mutations in the dysferlin gene. Dysferlin is involved in membrane repair and vesicle fusion through its 7 C2 calcium-binding domains, which mediate these calcium-dependent processes. It is currently considered an untreatable neuromuscular condition with a poor prognosis. The estimated incidence of this disease is 1 to 6.5 per 100,000 individuals. The primary clinical features of LGMD2B include proximal muscle weakness and elevated serum creatine kinase (CK) levels; occasionally, patients may present with elevated transaminase and hematuria. It is often misdiagnosed as polymyositis or liver disease. Herein, we report a case of LGMD2B initially presenting with elevated transaminase levels and hematuria.

**Patient concerns:**

A 30-year-old woman was found to have elevated transaminases and subsequent hematuria.

**Diagnoses:**

The patient was ultimately diagnosed with LGMD2B after muscle biopsy and genetic testing were performed.

**Interventions:**

In the patient’s first hospitalization, she was found to have elevated transaminase levels and untested CK levels, and no pathogenic findings were identified after a liver biopsy. Four years later, the patient was admitted to the Nephrology Department with gross hematuria. After hospitalization, serum CK levels were elevated. She was misdiagnosed with polymyositis and treated with oral prednisone; however, her condition did not improve, muscle strength declined, and hematuria persisted. Muscle biopsy and genetic testing were performed, and the patient was ultimately diagnosed with LGMD2B. Supportive therapy with coenzyme Q10, idebenone, and creatine monohydrate was initiated, and she was advised to avoid strenuous physical activity.

**Outcomes:**

Currently, the patient exhibits generalized muscle weakness, unstable walking gait, and urine positive (+++) for occult blood. Her muscle strength has gradually declined over the past 13 years.

**Lessons:**

LGMD2B initially presents with atypical clinical manifestations. In some cases, elevated transaminase levels can be the first manifestation of the disease; therefore, any unexplained elevated transaminase levels should prompt evaluation of underlying muscle diseases. A 13-year follow-up of a female patient demonstrated progressive muscle atrophy, emphasizing the importance of considering muscle diseases in patients with unexplained elevated serum transaminase levels. Subsequent dark brown urine and hematuria were likely caused by increased myoglobin levels and dysferlin deficiency in podocytes, which may be associated with minimal change nephropathy.

## 1. Introduction

Limb-girdle muscular dystrophy type 2B (LGMD2B) is an autosomal recessive myopathy induced by mutations in the *dysferlin* gene (*DYSF*), characterized by progressive limb weakness and elevated muscle enzyme levels.^[1]^ The estimated incidence of this disease is 1 to 6.5 per 100,000 individuals.^[2]^ Loss of ambulation occurred generally during the fourth decade.^[3]^ Mutations in the *DYSF* often lead to secondary membrane damage following mechanical and chemical stress, resulting in increased Ca^2+^ influx and subsequent mitochondrial aberrations.^[4,5]^ Impaired dysferlin-mediated membrane repair causes progressive myonecrosis.^[6]^ Myolemma damage often elevates serum creatine kinase (CK) and myoglobin levels, and excessive myoglobin may in turn cause kidney injury.^[7]^

The early symptoms of LGMD2B are often subtle, elevated transaminase and CK levels may be observed during routine physical examinations. Aspartate aminotransferase is present in the liver and other organs, including the cardiac and skeletal muscles, kidneys, and brain.^[8]^ The most common causes of elevated transaminase levels are nonalcoholic fatty liver disease and alcoholic liver disease. Less common causes include drug-induced liver injury, hepatitis B and C infections, and hereditary hemochromatosis, while rarer causes include alpha-1 antitrypsin deficiency, autoimmune hepatitis, and Wilson disease. Extrahepatic sources, such as thyroid disorders, celiac sprue, hemolysis, and muscle disorders, are also associated with mildly elevated transaminase levels.^[9]^ However, long-term follow-up data on patients with LGMD2B, especially those initially misdiagnosed due to atypical early biochemical findings, remain limited.

Herein, we report the case of a 30-year-old woman who initially presented with elevated transaminase and CK levels. The patient was misdiagnosed with liver disease and polymyositis (PM). Further muscle pathology and gene testing revealed a *DYSF* mutation. Ultimately, the patient was diagnosed with LGMD2B. A 13-year follow-up showed a gradual decline in muscle strength, and magnetic resonance imaging (MRI) of the lower-limb muscle revealed fatty replacement of the muscles. All analyses were conducted after the patient provided written informed consent. This case demonstrates that LGMD2B can be misdiagnosed as liver disease or PM during the early stages of the disease.

## 2. Case presentation

A 30-year-old woman was found to exhibit elevated transaminase levels during a preemployment medical examination (alanine transaminase [ALT]:119 U/L, normal range: 0 to 40 U/L; aspartate transaminase [AST]: 121 U/L, normal range: 5–34 U/L) without any symptom. The patient had no history of hepatitis, alcohol consumption, or drug abuse, and no history of hereditary or familial diseases.Tests for anti-mitochondrial, anti-Sjögren syndrome A (anti-SSA), anti-Sjögren syndrome B (anti-SSB), anti-Sm, and anti-smooth muscle antibodies were negative. Similarly, tests for anti-EBV and anti-cytomegalovirus antibodies were also negative. Given that elevated transaminase levels are often attributed to liver disease, the patient underwent a liver biopsy on October 29, 2009. Histopathological examination revealed sparse and swollen hepatocytes with minimal dot-like necrosis in the hepatic lobules; however, no piecemeal necrosis was found in the portal area. A small degree of lymphocyte infiltration was observed, without fibrotic tissue proliferation. The CK19 assay showed no bile duct abnormalities. Overall, the liver pathology did not indicate liver disease. The patient was prescribed polyene phosphatidylcholine (576 mg, 3 times daily; oral intake).

On August 21, 2012, the patient was hospitalized with dark brown urine following a high fever with shivering caused by a respiratory infection. At this time, serum CK levels were measured and found to be 4309 U/L (normal range, 20–200 U/L), CK-myocardial band (CK-MB) was 65 U/L (normal range, 0–25 U/L), and serum creatinine was 49 µmol/L (normal range, 41–73 µmol/L). On physical examination, the patient’s body temperature was 38.6 ℃. Muscle strength in all 4 limbs was grade 5, based on the Medical Search Council scale.^[10]^ Routine blood tests showed a normal white blood cell count, a neutrophil percentage of 85% (normal range, 40%–75%), hemoglobin of 126 g/L (normal range, 113–151 g/L), and a normal platelet count. Routine urinalysis showed erythrocytes at 3/4 high-power field, a deformity rate of 65%, positive urine protein (++), and elevated serum myoglobin levels (> 3000 μg/L (normal range, 10–70 μg/L). Tests for antinuclear antibodies, rheumatoid factor, and anti-Jo-1 antibodies were negative. Screenings for myositis-related antibodies, including anti-signal recognition particle (anti-SRP) antibody, anti-3-hydroxy-3-methylglutaryl coenzyme A reductase (anti-HMGCR) antibody, and myositis-specific autoantibodies, were also negative.

**Figure 1.**
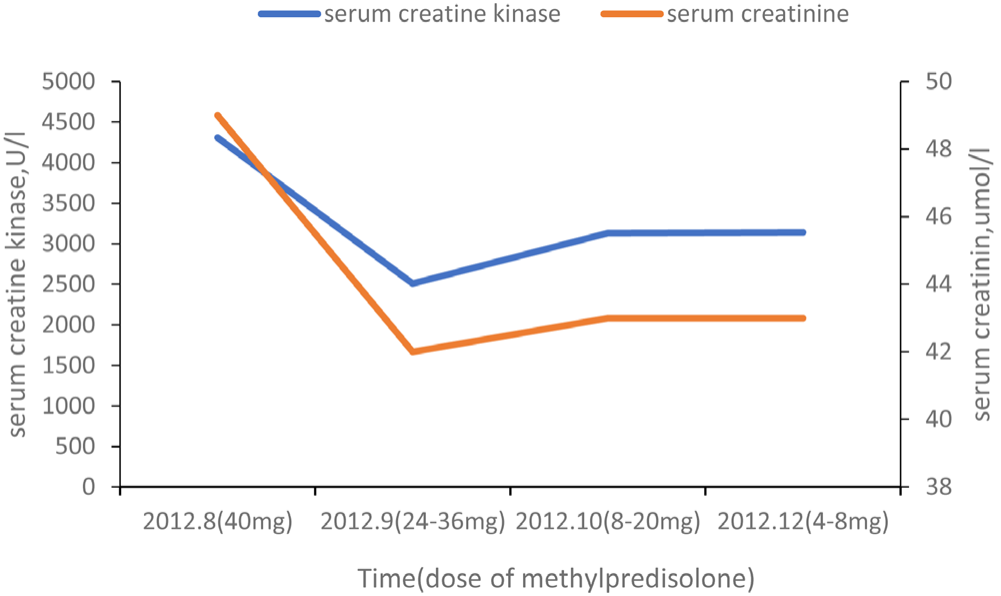
Changes in serum CK and creatinine levels during methylprednisolone use. CK = creatine kinase.

The patient received sulbactam and cefoperazone sodium injection (3 g every 12 hours, intravenous drip). The body temperature and white blood cell count returned to normal after 3 days of antibacterial therapy. Routine urinalysis revealed 20 to 25 erythrocytes per high-power field, a deformity rate of 80%, 2 erythrocyte casts per high-power field, positive urine protein, and a CK level of 4419 U/L. Electromyography (EMG) showed myogenic changes. Based on the high CK levels and abnormal EMG findings, the patient was diagnosed with suspected PM and treated with methylprednisolone (40 mg daily). A reexamination 1 month later showed CK at 1993 U/L; however, 2 months later, it increased again to 2574 U/L. Although the CK level decreased, the patient’s muscle strength gradually declined during treatment with prednisone. Methylprednisolone dose was reduced by 1 tablet per week until discontinuation (Fig. 1).

**Figure 2.**
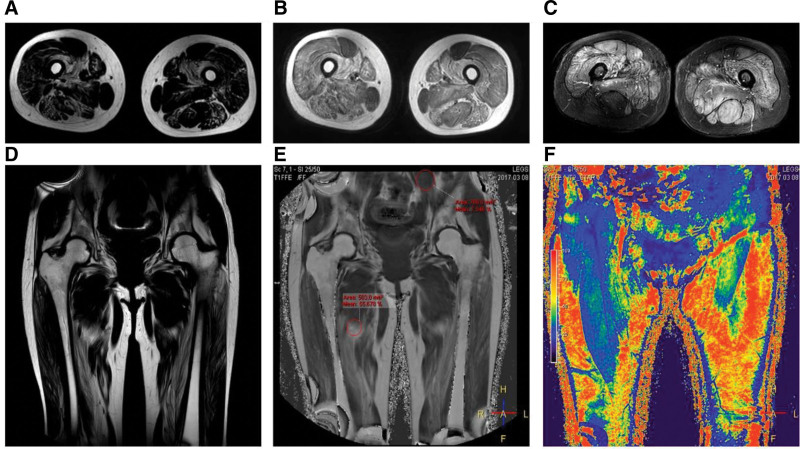
(A) T2WI-weighted image showing diffuse fatty infiltration of the lateral femoral muscle and intermediate femoris muscle. (B) T1WI-weighted image showing diffuse edema of the lateral femoral muscle and intermediate femoris muscle. (C) FLAIR sequence. (D) T2WI-weighted image. (E) T1WI-weighted image showing diffuse edema of the thigh muscles. (F) Spectrum sequence showing diffuse lipidification of the thigh muscles.

**Figure 3.**
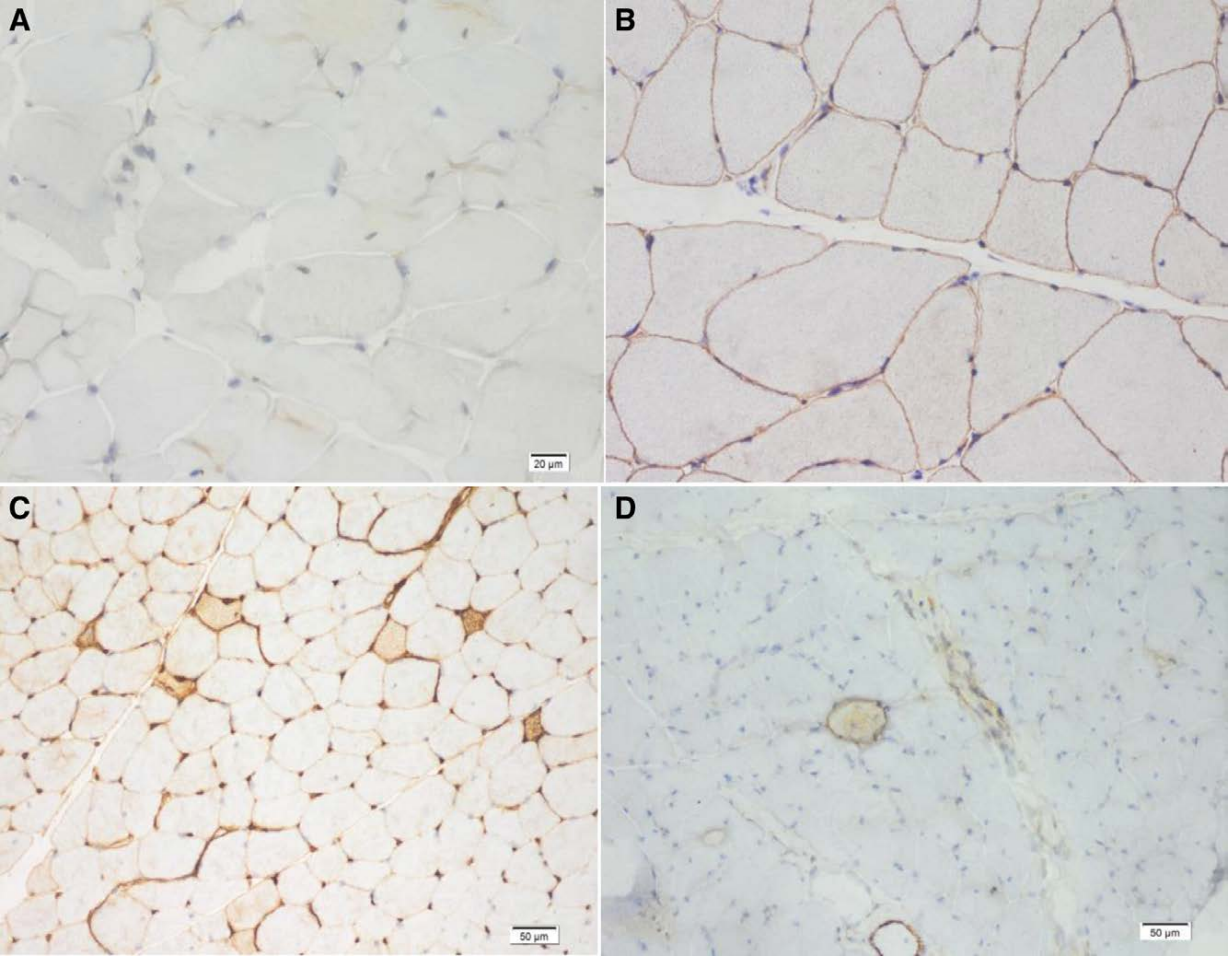
Immunohistochemistry of the muscle biopsy. (A) Dysferlin was totally absent in the sarcolemmal membrane, 40×. (B) Dysferlin positive control, 40×. (C) Many degenerated myofibers showing variation in size and positivity for C5b9, 20×. (D) Positivity for major histocompatibility complex class I, 20×.

**Figure 4.**
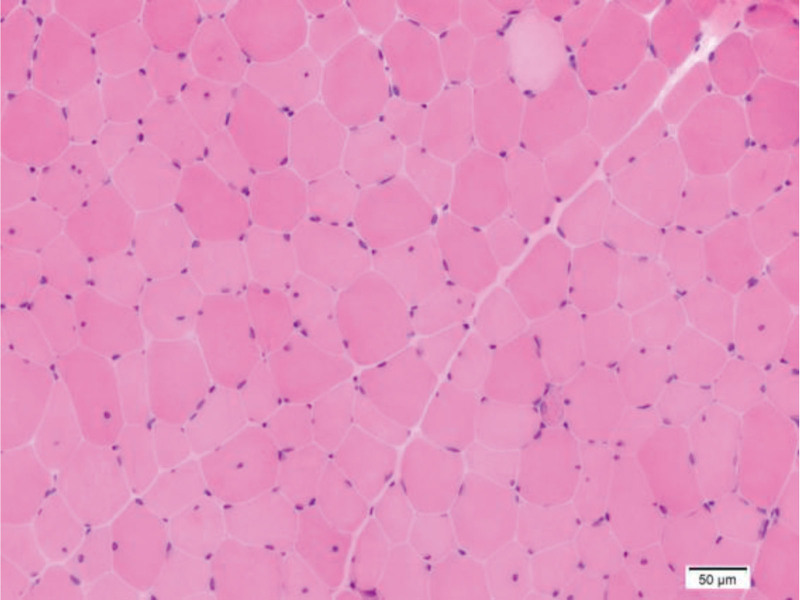
Degenerated myofibers variation in fiber size, H&E stain, 20×.

**Figure 5.**
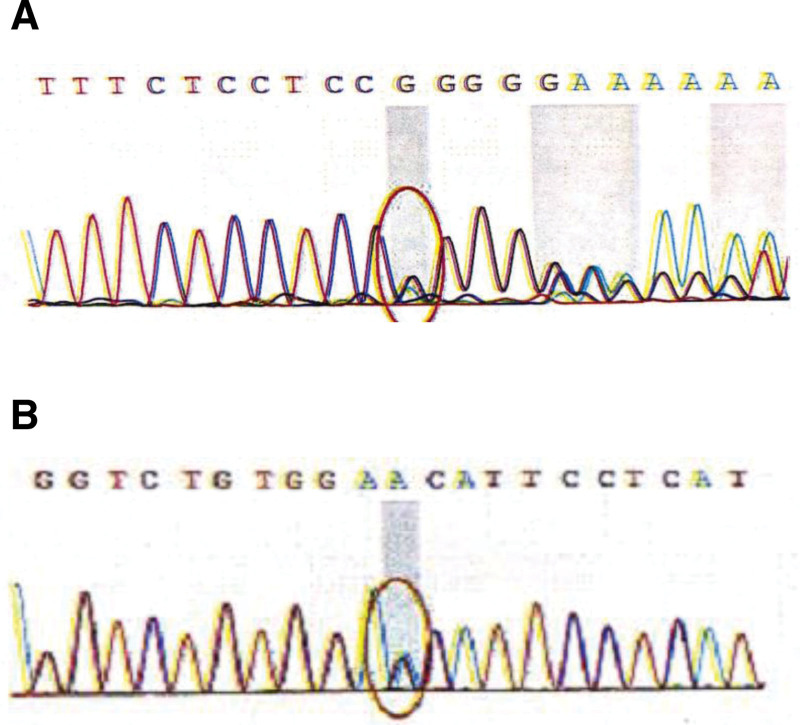
(A) Heterozygous mutation of c.1560delT at chr2:71766353. (B) Heterozygous mutation of c.3093G > T at chr2:71797430.

As the patient’s condition worsened following prednisone administration, further immunological tests, MRI, and muscle pathology examinations were performed. Examination results for myositis antibodies, anti-SRP antibodies, anti-HMGCR antibodies, and myositis-specific autoantibodies were negative. MRI of the bilateral lower-limb muscles revealed edema and solidification in the thigh muscles and cingulum membri inferioris (Fig. 2). Immunohistochemistry demonstrated positive staining for C5b-9 and major histocompatibility complex class I on the myolemma, but negative staining for dysferlin, suggesting dysferlinopathy (Fig. 3). Hematoxylin and eosin staining revealed endomysial fibro-fatty changes (Fig. 4). Although dysferlin is not a routine test on immunohistochemistry, among the currently known common diseases that cause progressive muscle weakness, defects in the proteins dystrophin and dysferlin are both common causes, so the dysferlin immunohistochemistry was conducted for the patient. Subsequently, a blood sample was obtained for comprehensive genetic testing (using the Agilent exon chip capture and high-throughput sequencing method) for SMT-01 neuromuscular disease, focusing on *DYSF*. The genetic analysis identified compound heterozygous mutations – c.1560delT (deletion) and c.3093G > T (guanine-to-thymine substitution) – resulting in amino acid variations of p.P520fs (frameshift mutation) and p.W1031C (tryptophan-to-cysteine) (Fig. 5). The patient had 2 heterozygous mutations (c.1560delT and c.3093G > T), respectively, inducing a frameshift mutation and a substitution, and the 2 mutations are on different gene alleles.Based on these findings, the patient was diagnosed with LGMD2B.

Supportive therapy with coenzyme Q10 (100 mg, once daily), idebenone (150 mg, 3 times daily), and creatine monohydrate (8 g, 3 times daily) was initiated. Serum CK levels were reevaluated monthly and ranged between 6000 and 8000 U/L. After 3 months, no substantial improvement was observed, and the therapy was discontinued. Notably, the patient reported a substantial reduction in CK levels during pregnancy in 2015, suggesting a possible influence of hormonal changes. On November 7, 2019, muscle strength was grade 5 in all limbs. Routine urinalysis revealed negative urine protein, positive occult blood (++), and urine erythrocyte was 6 to 8 high-power field. On May 14, 2020, CK was 6190 U/L, and CK-MB was 111 U/L. From 2020 to December 30, 2024, the patient was followed up annually to examine CK levels and muscle strength. The CK ranged between 2509 and 7539 U/L, ALT between 63 and 134 U/L, and AST between 63 and 109 U/L. Serum creatinine decreased from 41 to 32 µmol/L (normal range, 41–73 µmol/L). On December 30, 2024, the patient exhibited progressive weakness, had difficulties climbing stairs and squatting, required external assistance to rise from the squatting position, and exhibited an unstable walking gait. Although gross hematuria was absent, urinalysis showed occult blood (+++), with no erythrocytes observed under the microscope. Muscle strength was grade 3 + in the left thigh, grade 3 in the right thigh, and grade 4 + in both upper limbs. Serum CK was 5538 U/L, ALT was 84 U/L, and AST was 84 U/L. The changes in serum biochemical indicators during the follow-up period are presented in Table 1.

**Table 1:** Changes in serum biochemical indicators during the follow-up period.

Time	ALT (U/l) (normal range:0–40)	AST (U/l) (normal range:5–34)	CK (U/l) (normal range:20–200)	Creatine (µmol/l) (normal range:41–73)	Muscle strength level (grade:0–5)	Medication and other information description
2012.8	134	115	4309	49	5	Methylpredisolone 40 mg/qd
2012.9	84	54	1993	42	4+	Methylpredisolone 24–36 mg/qd (reduce 4 mg/wk)
2012.10	63	105	2574	43	4+	Methylpredisolone 8–20 mg/qd (reduce 4 mg/wk)
2012.12	111	63	3142	43	4+	Methylpredisolone-4–8 mg/qd (reduce 4 mg/2 wk)
2013.12	87	67	4263	40	5	–
2014.12	93	88	5234	39	5	–
2015.6	98	86	4325	40	5	–
2016..6	103	82	4982	36	4+	–
2017.12	127	93	5973	41	4	–
2018.12	122	109	7539	35	4	–
2019.12	92	90	6273	37	4	–
2020.11	93	97	7427	39	4	–
2021.11	84	78	6007	37	3+	–
2022.12	64	76	4350	36	3+	–
2023.11	69	78	5706	30	3+	–
2024.12	84	84	5538	36	3+	–

ALT = alanine transaminase, AST = aspartate transaminase, CK = creatine kinase.

## 3. Discussion

LGMD2B is a typical phenotype of dysferlinopathy caused by mutations in *DYSF*. *DYSF* is located on chromosome 2p12–14, comprises 55 exons spanning approximately 150 kb of genomic DNA, and encodes a 237-kDa protein.^[11]^ It is an autosomal recessive disease characterized by lipidification of the proximal limb muscles and progressive muscular atrophy. The mean serum CK level in patients with LGMD2B is 4342 ± 3518 U/L.^[12]^ MRI of the thighs typically shows edema of the semimembranosus, semitendinosus, and biceps femoris muscle, along with fatty infiltration.^[13,14]^ In this case, the patient’s CK levels ranged from 1993 to 7539 U/L. Muscle pathology showed variability in fiber size, with both necrotic and regenerating fibers, and the absence of dysferlin expression confirmed the diagnosis. Electrophysiological, radiological, and histopathological findings were consistent with this diagnosis. The patient had 2 heterozygous mutations – c.1560delT and c.3093G > T – that induced a frameshift mutation and substitution, respectively. Previous studies have reported various *DYSF* mutation types, including missense, nonsense, frameshift, in-frame deletions, intronic mutations, and exon shuffling. However, no correlation between genotypes and phenotypes was found, and no mutation hotspots have been identified.^[15]^

The patient reported here initially presented with elevated transaminase levels and was first considered to have liver disease, followed by a misdiagnosis of PM. The initial clinical manifestations of LGMD2B are variable and may include muscle weakness, elevated CK, and increased AST levels. In rare cases, patients may present with dark brown urine as the initial manifestation. In patients with unexplained transaminase elevation or muscle weakness, the CK levels should be measured to determine whether the condition is caused by myopathy. Liver disease is the most common cause of elevated serum transaminase levels. Less common causes include drug-induced liver injury, hepatitis B and C infections, and hereditary hemochromatosis, while rarer causes include alpha-1 antitrypsin deficiency, autoimmune hepatitis, and muscle disorders. In this case, the patient was initially misdiagnosed with liver disease. Therefore, unexplained elevations of transaminase levels require testing for CK and other factors to rule out extrahepatic causes, particularly muscle diseases. Elevated CK levels associated with LGMD2B are often misdiagnosed as PM. PM is a CD8 + T cell-mediated autoimmune disease that primarily affects the proximal muscles of the limbs.^[16]^ Because of misdiagnosis with multiple myositis and subsequent treatment with prednisone, the patient’s condition worsened, and muscle strength declined.

Dysferlin protein is expressed in both cardiomyocytes and skeletal muscle cells and is involved in the repair of cell membranes.^[17]^ Muscular dystroproteinopathy primarily manifests as skeletal muscle disease, and apparent cardiomyopathy is relatively uncommon. In addition to the progressive skeletal muscle weakness, the patient in this study only exhibited an increase in CK-MB levels but no symptoms related to dilated cardiomyopathy or heart failure. Similarly, a previous study^[18]^ reported dilated cardiomyopathy in only 2 of 7 patients with LGMD2B. The data indicated that dysferlin deficiency and disorders of the Z-line protein and transcription factors, especially under mechanical stress, could lead to cardiomyopathy. This difference in clinical manifestations between the skeletal and cardiac muscles may reflect the different mechanical stresses borne by these tissues. In our patient, the CK-MB elevation may indicate the presence of subclinical myofascial instability, consistent with previous reports, but without progression to significant systolic dysfunction. Genetic testing was conducted using DNA extracted from peripheral blood. The sequencing chromatogram showed that the sequencing depths of the 2 variants (c.1560delT and c.3093G > T) were 355/307 (0.46) and 233/228 (0.49), respectively, consistent with a heterozygous pattern rather than with a chimeric pattern. Therefore, we believe that tissue-specific phenotypes can be better explained by the reasons mentioned above rather than genetic chimeras. Accurate diagnosis is crucial for patients with LGMD2B, particularly in the early stages, as avoiding inappropriate prednisone use can prevent additional muscle damage. So we suggest every progressive weakness patient with elevated CK should wait for gene exam or immunohistochemical examination of skeletal muscle before steroid use.

During the follow-up period, no gradual increase in CK levels was observed; however, creatinine levels decreased, which may be associated with muscle steatosis and reduced muscle mass. This finding is consistent with the observed decline in muscle strength, suggesting that serum creatinine may be an indicator of disease severity.

Other clinical manifestations were dark brown urine and elevated serum myoglobin levels. These symptoms must be investigated, as they may indicate rhabdomyolysis. The presence of red blood cells in the urine also suggests possible kidney injury. Under physiological conditions, plasma myoglobin concentrations are extremely low (0–0.003 mg/dL). When over 100 g of skeletal muscle is damaged, circulating myoglobin levels exceed the protein-binding capacity of the plasma and may precipitate in the glomerular filtrate. Excess myoglobin can subsequently lead to renal tubular obstruction, direct nephrotoxicity, and acute tubular necrosis.^[19]^ Izzedine et al reported that dysferlin loss in podocytes led to mitochondrial cytopathy with neurodegeneration and brain iron accumulation, characterized by proteinuria and microscopic hematuria. Similarly, dysferlin deficiency in podocytes is associated with minimal change nephropathy.^[20]^ In this case, the patient initially presented with gross hematuria at presentation, followed by persistent microscopic hematuria, indicating possible renal damage. Although the patient’s abnormal indicators, such as elevated CK levels, were identified following a respiratory tract infection accompanied by changes in urine color, proteinuria, and hematuria, a renal biopsy was not performed for the following reasons: the patient exhibited unexplained elevations in transaminases, CK, and CK-MB, with particularly marked increases in CK and CK-MB. Hematuria and proteinuria were therefore considered possible manifestations of the systemic involvement of the primary disease. After some treatments, a downward trend in hematuria and proteinuria was observed, suggesting that the renal lesions were effectively controlled. Because renal biopsy is an invasive procedure associated with potential risks such as bleeding, infection, and arteriovenous fistula formation, we determined that the expected benefits did not significantly outweigh these risks. However, during this process, the patient’s condition was closely monitored, and no progressive deterioration of renal function was observed.

Current research for dysferlinopathy therapy focuses on symptomatic treatment, antisense-mediated exon skipping, myoblast transplantation, and gene editing. In a mouse model of severe LGMD2B, treatment with ezetimibe completely prevented clinical signs of ambulatory dysfunction.^[21]^ Noemi De Luna et al reported that treatment with vitamin D3 increased expression of dysferlin in vitro.^[22]^ Glucocorticoids can destabilize the dysferlin-deficient muscle cell membrane and further compromise their repair. Long-term glucocorticoid use may induce the atrophy and necrosis of type II muscle fibers and lead to poor muscle regeneration.^[23]^ Vamorolone, unlike prednisolone, has been shown to stabilize dysferlin-deficient sarcolemmal membrane and improve repair of dysferlin-deficient mouse muscle fibers following focal or eccentric contraction-induced injury.^[24]^ Additionally, Gushchina LV et al reported that short-term rhMG53 treatment could improve dysferlin-deficient myopathy by increasing sarcolemmal membrane integrity.^[25]^ Antisense oligonucleotides are short, synthetic, single-stranded oligonucleotides that can bind to specific regions of mRNA or pre-mRNA to modulate gene expression through several distinct mechanisms, depending on their chemical structure and target sequence.^[26]^

Although LGMD2B is a known disease, the uniqueness of this case lies in the 13-year longitudinal follow-up, which provides valuable clinical data. The patient initially presented with high fever and chills caused by a respiratory tract infection, accompanied by brownish urine. This progression from misdiagnosis to confirmed diagnosis demonstrates the critical importance of routine CK testing in cases of unexplained elevated transaminase levels. Notably, the patient’s condition deteriorated after prednisone administration before the underlying cause was identified. Additionally, we recorded the dynamic changes in CK levels during pregnancy, providing a unique perspective on the interaction between environmental and genetic factors in disease expression.

## 4. Conclusions

This case of LGMD2B demonstrates that the clinical presentation of the disease is atypical. Muscle weakness, elevated transaminase and CK levels, and dark brown urine with erythrocytes may be the initial manifestations. Skeletal muscle damage may increase serum transaminase levels; therefore, CK testing is essential in cases of unexplained transaminase elevation to determine whether the condition is caused by myopathy. We suggest that genetic myopathy must be ruled out before establishing a diagnosis of liver disease, suspected PM, or unexplained dark brown urine. Early and accurate diagnosis of LGMD2B is crucial, as timely recognition of muscle involvement can prevent misdiagnosis and inappropriate glucocorticoid administration. In this case, the decline in creatinine level was consistent with the decrease in muscle strength, suggesting that creatinine may be an indicator for evaluating disease severity.This case highlights the need for further studies to validate serum creatinine as a potential indicator of disease severity and to better understand the renal implications of dysferlin deficiency.

## Acknowledgments

We would like to thank Editage (www.editage.com) for English language editing.

## Author contributions

**Conceptualization:** Zhenhua Ji.

**Data curation:** Zhenhua Ji, Ying Yang.

**Investigation:** Hua Zheng.

**Writing – original draft:** Hua Zheng.

**Writing – review & editing:** Dan Li, Zhong Li, Xinxin Guo.
